# Non-genetic influences on lipoprotein(a) concentrations

**DOI:** 10.1016/j.atherosclerosis.2022.04.006

**Published:** 2022-05

**Authors:** Byambaa Enkhmaa, Lars Berglund

**Affiliations:** a Department of Internal Medicine, School of Medicine, University of California Davis, Davis, CA, USA; b Center for Precision Medicine and Data Sciences, School of Medicine, University of California Davis, Davis, CA, USA

**Keywords:** Lp(a) plasma level, Diet, Saturated fat, Physical activity, Hormones, Kidney disease, Liver disease

## Abstract

An elevated level of lipoprotein(a) [Lp(a)] is a genetically regulated, independent, causal risk factor for cardiovascular disease. However, the extensive variability in Lp(a) levels between individuals and population groups cannot be fully explained by genetic factors, emphasizing a potential role for non-genetic factors. In this review, we provide an overview of current evidence on non-genetic factors influencing Lp(a) levels with a particular focus on diet, physical activity, hormones and certain pathological conditions. Findings from randomized controlled clinical trials show that diets lower in saturated fats modestly influence Lp(a) levels and often in the opposing direction to LDL cholesterol. Results from studies on physical activity/exercise have been inconsistent, ranging from no to minimal or moderate change in Lp(a) levels, potentially modulated by age and the type, intensity, and duration of exercise modality. Hormone replacement therapy (HRT) in postmenopausal women lowers Lp(a) levels with oral being more effective than transdermal estradiol; the type of HRT, dose of estrogen and addition of progestogen do not modify the Lp(a)-lowering effect of HRT. Kidney diseases result in marked elevations in Lp(a) levels, albeit dependent on disease stages, dialysis modalities and apolipoprotein(a) phenotypes. In contrast, Lp(a) levels are reduced in liver diseases in parallel with the disease progression, although population studies have yielded conflicting results on the associations between Lp(a) levels and nonalcoholic fatty liver disease. Overall, current evidence supports a role for diet, hormones and related conditions, and liver and kidney diseases in modifying Lp(a) levels.

## 1. Introduction

**Fig. 1.**
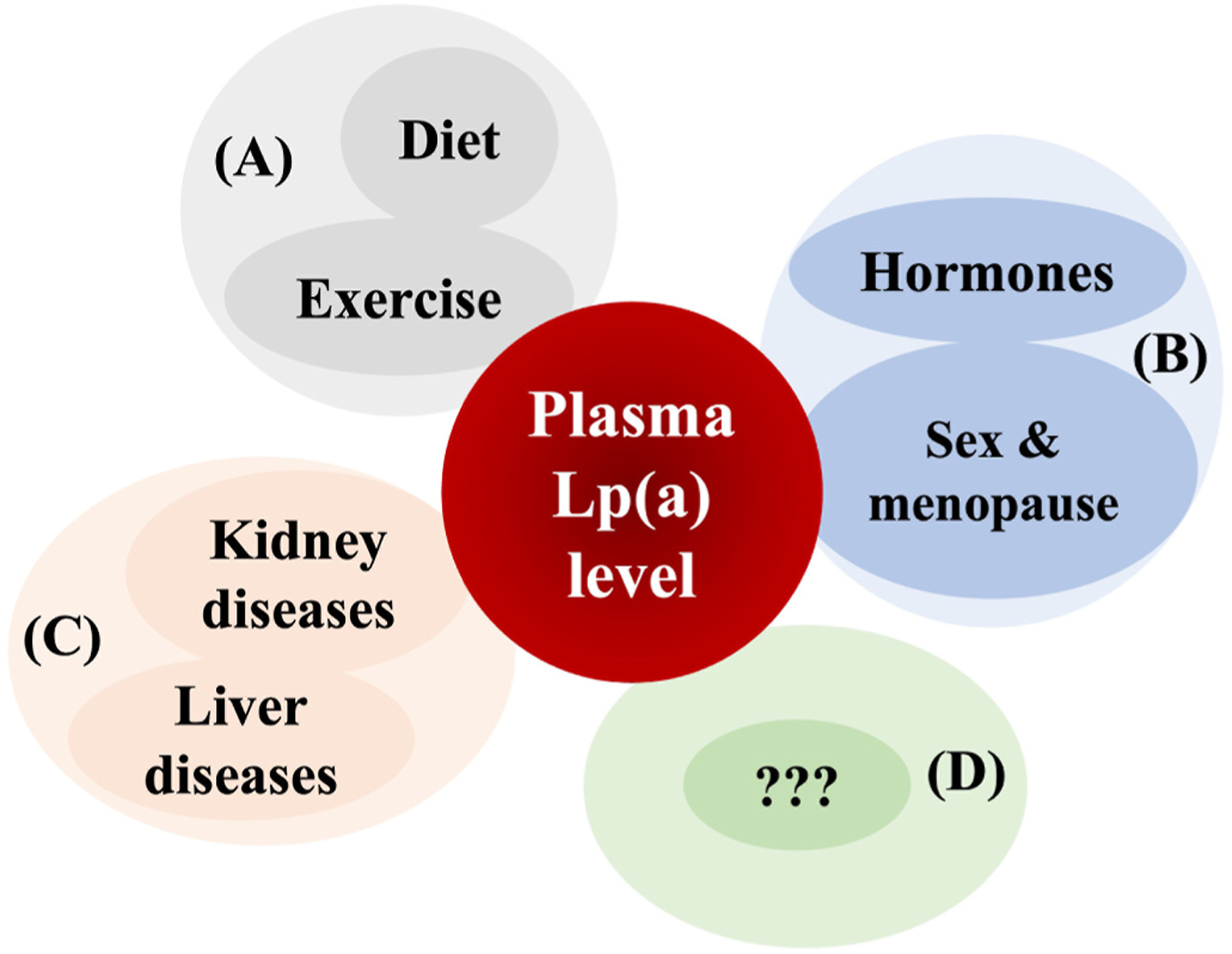
Non-genetic factors influencing plasma Lp(a) levels. Although plasma Lp(a) levels are mostly genetically determined, some evidence suggests that non-genetic factors may also influence Lp(a) levels. These include lifestyle factors such as diet. In particular, reduction in dietary saturated fat intake and exercise (A), hormones and associated conditions such as menopause (B) and chronic conditions such as liver and kidney diseases that impact synthesis and catabolism of Lp(a) (C). Other factors with a potential to influence Lp (a) levels remain to be identified (D).

It is well established that elevated Lp(a) levels are an independent casual risk factor for cardiovascular diseases (CVD), including coronary artery disease (CAD), myocardial infarction (MI), and aortic valve stenosis [1]. This is discussed in detail by Arsenault and Kamstrup in another review of this series [2]. In addition, recent studies indicated a role also in heart failure [3]. Lp(a) levels are strongly determined through genetic variants in the *LPA* gene, particularly by a size polymorphism in apolipoprotein(a) [apo(a)] as reviewed by Coassin and Kronenberg [4]. The present review will focus on the roles of non-genetic factors such as diet and physical activity (PA) and the influence by sex and hormones (Fig. 1). We will also summarize evidence on pathological conditions that modify Lp(a) levels, including kidney and liver diseases, emphasizing the magnitude and directionality of their effects as pertinent to cardiovascular risk as well as the apo(a) size polymorphism (for a summary, see Box 1).

## 2. Non-genetic factors and Lp(a) levels

### 2.1. Diet

One of the first human clinical trial evidence that diet may modulate Lp(a) concentration was reported by Hornstra et al. [5] who observed a 10% reduction in Lp(a) concentration with a palm-oil enriched diet compared to a control Dutch diet. In further support of an impact of fat quality, a 23% increase in Lp(a) concentration was seen in response to a high oleic-acid diet with ~10% compared to a diet with 19% of calories from saturated fatty acids (SFA) [6]. Notably, LDL-C levels decreased by 17%. Replacement of SFA with *trans*-monounsaturated fatty acids resulted in an even higher increase (73%) in Lp(a) level. Further, compared to a control high-SFA diet, diets lower in SFA and proportionately higher in monounsaturated fatty acids (MUFA) or polyunsaturated fatty acids (PUFA) tended to increase Lp(a) but the change was not significant [6].

The two DELTA (Dietary Effects on Lipoproteins and Thrombogenic Activity) trials were the first randomized multicenter dietary studies in participants with differing metabolic profiles [7,8]. The DELTA 1 trial recruited healthy participants and demonstrated that lowering dietary SFA intake from 16% to 5% of calories with a proportionate increase in complex carbohydrate (CHO) increased Lp(a) levels by ~15% [7]. The DELTA 2 study undertaken in participants with a high-risk metabolic profile showed that isocaloric replacement of SFA with complex CHO or MUFA increased Lp(a) levels by 20% and 11%, respectively [8]. In both DELTA trials, as expected, LDL-C was reduced by 7–11%. Collectively these two DELTA trials demonstrated opposite changes in Lp(a) and LDL-C in response to dietary SFA replacement [7,8]. Other studies have reported similar findings replacing SFA with MUFA, PUFA, or a combination of MUFA and PUFA [9–11].

A large randomized crossover feeding trial in adults with prehypertension or stage 1 hypertension (The Omni Heart Trial) tested differences in Lp(a) responses to DASH (Dietary Approaches to Stop Hypertension)-style diets differing in macronutrient content (either rich in CHO, protein, or unsaturated fat) and analyzed the responses by race [12]. All three diets increased Lp(a) level by ~8–18% compared to baseline after six weeks; however, the diets rich in unsaturated fats increased Lp(a) less than diets rich in CHO or protein and greater changes were observed in Black participants than in White participants [12]. In this cohort, LDL-C was reduced by 12–14 mg/dL across all three test diets [13].

A few studies have examined the effect of low-fat, high-CHO (LFHC) diets compared to high-fat, low-CHO (HFLC) diets on Lp(a). Compared to a HFLC diet, a LFHC diet increased Lp(a) levels by ~12% and lowered LDL-C by ~7 mg/dL [14]. This study also showed increases in oxidized phospholipids (OxPL) per apolipoprotein (apo)B or apo(a) with the LFHC diets [14]. Diet-induced changes in Lp(a) concentration were strongly correlated with changes in OxPL per apoB. Lp(a) is the primary carrier of circulating OxPL and as the OxPL content is hypothesized to mediate its atherogenicity further studies on the impact of diet are warranted [15,16]. The topic on OxPLs carried on Lp(a) is discussed in detail by Koschinsky and Boffa in this review series [17].

In a recent randomized feeding trial, after an initial 10–14% weight loss, three maintenance diets containing 20% protein and differing 3-fold in CHO and SFA as a proportion of energy were consumed for 20 weeks [18]. While Lp(a) levels decreased by ~15% in the low-CHO/high SFA group, no changes were observed in the moderate-CHO and high-CHO groups [18]. Collectively, there is strong documentation that short-term dietary interventions to reduce SFA intake result in an increase in Lp(a) levels of 9–23%, while at the same time decreasing LDL-C levels by 7–17%, depending on the type of replacement strategy and cohort characteristics.

The question whether fasting *versus* nonfasting conditions would impact Lp(a) levels was recently addressed and similar Lp(a) concentrations under both conditions were reported [19]. A larger dietary change in the Lp(a) concentration was reported in a n = 1 case study of a male physician with a very high Lp(a) level who undertook changes in dietary CHO consumption [20]. Lp(a) levels varied considerably depending on the diet regimen, with a decrease during a very-low CHO ketogenic diet followed by an increase in the Lp(a) level after two weeks of a very high-CHO (400 g/day) diet, again being reduced after three weeks of restarting the very-low CHO ketogenic diet [20]. These observations are in line with the notion that substitution of SFA with unsaturated fat, but not with CHO, is a preferable regimen in terms of Lp(a) levels [12]. The rapid onset of these changes indicates a flexible regulation of Lp(a) levels in response to diet modulation.

On the other hand, some studies have not found an increase in Lp(a) levels with a reduction of dietary SFA. For example, a 12-week intervention with a Mediterranean-style low-glycemic-load diet with reduced energy intake from CHO and fat, replaced by protein, lowered Lp(a) concentration by ~50% in women with the metabolic syndrome (MetS) [21]. Furthermore, a randomized crossover controlled feeding trial among overweight and obese participants found a modest but significant decrease in Lp(a) levels when a low-fat diet (24% total fat; 7% SFA) was compared to an average American diet (AAD) (34% total fat; 13% SFA) [22]. More recently, a 6-week randomized crossover controlled feeding study among at risk individuals reported an ~11% reduction in Lp(a) levels with a PUFA-enriched diet, while no change in Lp(a) levels was seen with a MUFA-enriched diet [23]. The contrasting observations in these trials *versus* the other trials with regard to Lp(a) responses to SFA reduction (a decrease *versus* an increase) need to be further explored, although differences in Lp(a) measurement methodology, test diets or cohort characteristics might contribute. Notably, as the vertical auto profile (VAP) method uses an ultracentrifugation technique and relies on Lp(a) cholesterol rather than quantification of Lp(a) concentrations, a potential overlap of the Lp(a) fraction with other lipoprotein fractions cannot be excluded using this approach [22–25].

Beyond macronutrient changes, the potential effects on Lp(a) levels by diets enriched with nuts (walnuts [26], pecans [27] or almonds [28–31]) have been explored. While a modest reduction in levels (6–15%) was seen in randomized trials using diets enriched with walnuts (41–56 g/day) [26] or pecans (72 g/day) [27], studies on almond-enriched diets report inconsistent findings [28–31]. Further studies are needed to establish a role in particular for almonds with regard to dietary modulation of Lp(a) levels.

Regarding the role of alcohol consumption in Lp(a) level, an analysis of a large European American sample found no association between alcohol consumption and Lp(a) level [32], while a large study in middle-aged Chinese individuals reported a slight decrease in Lp(a) levels in male heavy drinkers compared with abstainers [33]. In intervention studies using red wine, no change in patients with carotid atherosclerosis [34] or a decrease in men at high risk for CVD have been reported for Lp(a) levels [35].

**Fig. 2.**
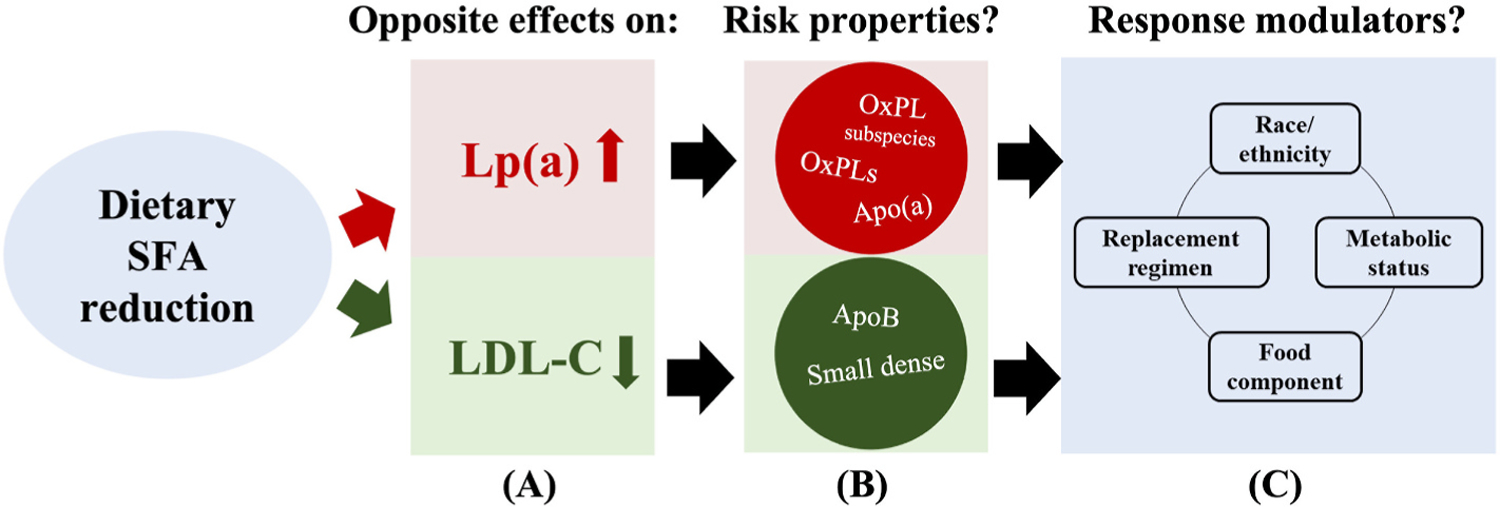
Opposite effects of reducing dietary saturated fat intake on Lp(a) and LDL-C concentrations and modulation of their risk mediating properties as well as impact by other factors. Reduction in dietary saturated fatty acid (SFA) intake can increase Lp(a) concentrations while inducing a consistent clinically meaningful reduction in LDL-C concentrations (A). Although the impact of dietary SFA reduction on LDL-C and its properties is well studied, limited data is available on its impact on Lp (a)s unique properties such as oxidized phospholipids (OxPLs) concentration or subspecies composition and any modulatory role by the apo(a) size polymorphism (B). Whether the responses to dietary SFA reduction in Lp(a) concentrations and properties would differ by an individual’s racial/ethnic background or metabolic burden and SFA replacement regimens or other food components in the diet remain to be established (C).

As the present format does not permit an in-depth analysis of the impact of nutrients on Lp(a), a more detailed summary that included a tabulation of such studies was recently published [36]. However, in summary, although the evidence from randomized controlled clinical trials during the last three decades on the dietary modulation of Lp(a) level is not fully consistent, an increasing body of evidence indicates that reductions in dietary SFA intake result in an increase in Lp(a) levels. The SFA replacement choice (CHO, MUFA, PUFA, or protein) and certain food/drink types (and the amount) in the diet beyond its macronutrient composition may also contribute to modulate Lp(a) levels. Notably, a dietary SFA reduction consistently decreased LDL-C, resulting in an opposite pattern compared to Lp(a) (Fig. 2). As proinflammatory and proatherogenic OxPLs may shuttle between Lp(a) and LDL-C particles, the diet-induced opposing changes in OxPLs’ plasma carriers merit further investigation and will help adopt precision nutrition approaches to reduce CVD risk.

### 2.2. Physical activity, exercise, and cardiorespiratory fitness

A potential role of PA and exercise in the modulation of Lp(a) levels has attracted interest. An early report of a Lp(a) decrease of ~22% in healthy young- and middle-aged men after an 8-day cross-country skiing regimen (equivalent to a 10 h of heavy PA/day) [37] indeed suggested an impact of PA. However, these results have been challenging to confirm as several studies have failed to find an association between Lp (a) levels and PA level or cardiorespiratory fitness [38–41]. Moreover, Lp(a) levels did not differ significantly between male athletes and sedentary controls [42–44]. Also, a prospective data in postmenopausal women did not find any influence of exercise alone on Lp(a) levels [45].

Neither has any significant impact by PA on Lp(a) levels been documented in short- or long-term interventional and prospective studies [46–49]. Thus, while an intensive 4-year individualized risk reduction program, recommending a healthy diet, increased PA and an individualized endurance training program in men and women with CAD improved the overall lipid profile, reduced body weight, increased exercise capacity and reduced dietary fat intake, there was no change in the Lp(a) concentration [48]. More recently, although an 8-month study to increase PA in middle-aged men and women with one or more traditional CVD risk factors reduced LDL-C, and increased HDL-C and proprotein convertase subtilisin/kexin type 9 (PCSK9) levels, the mean Lp(a) concentration was not significantly affected [50].

In contrast, some observational studies in younger populations report an association between PA and Lp(a) levels. Among Finnish children and young-adults (9–24 years old), Lp(a) levels were inversely correlated with leisure time PA with a dose-response manner [51]. Also, in young children and adolescents with type 1 diabetes mellitus, physical fitness was inversely associated with Lp(a) levels [52]. Furthermore, in younger men (23–33 years old), Lp(a) levels were higher and positively associated with the maximum aerobic capacity in long-distance runners and body builders with regular prolonged high-level exercise training compared to sedentary men [53]. In previously sedentary younger men and women (median age: <40 years), an intensive 9-month long-distance running exercise training program significantly increased Lp(a) levels with a nearly 2-fold increase in both men and women who completed a half-marathon [54].

Among men and women with type 1 and type 2 diabetes mellitus, Lp (a) concentration decreased (−13%) among those with higher baseline values (>30 mg/dL) after a 3-month individualized aerobic exercise program [55]. The change in Lp(a) levels was inversely correlated with baseline levels. Similarly, a small study in obese men and women with type 2 diabetes mellitus reported a significant decrease in Lp(a) levels following a 12-week low-intensity resistance training [56].

In summary, most of the available evidence suggests that PA, intensive exercise training, increases in exercise or cardiorespiratory fitness have no or minimal impact on Lp(a) concentration, while significantly influencing concentrations of other lipids and lipoproteins. However, results of some studies, particularly those in younger or diabetic populations, deviate from this and suggest a possible Lp(a)-modulating effect by a prolonged high-level exercise training, aerobic exercise or low-intensity resistance training. Nevertheless, the magnitude of exercise-induced changes in Lp(a) levels has generally been modest and any impact related to major genetic regulators of Lp(a) concentration such as the apo(a) size polymorphism has not been addressed. Additionally, the lack of a control group in some studies [54–56] may raise concerns about the quality of data as studies have suggested presence of a modest intra-individual temporal variability in mean Lp(a) levels [57]. Therefore, more studies with appropriate control groups are needed taking potential confounders such as apo(a) sizes and assay methodology into account.

## 3. Sex-specific differences and hormones

### 3.1. Sex-specific differences

While many studies across population groups (Blacks and Whites [58], Hungarians [59], Germans, Ghanaians, and Sans [60], Caucasians [61], Tibetans, Koreans, Chinese, Nigerians, and Belgians [62], Blacks in the Seychelles [63] or Italians [64]) have found no sex-specific differences in Lp(a) levels, some studies report higher Lp(a) levels in females than males. Thus, among children and adolescents, Lp(a) levels were significantly higher in girls than in boys for both Blacks and Whites [65] as well as for Arabs [66]. Another study reported higher Lp(a) levels in women than in men for Europeans [63] and Japanese [67], but not for Blacks in the Seychelles [63]. Addressing the potential influence of CAD familial predisposition on such findings, Barra et al. [68] demonstrated no significant difference in Lp(a) levels between healthy teenage brothers and sisters with a positive parental history of premature MI. In Europeans with CAD, a 2-fold higher Lp(a) level was observed in women compared to men after adjusting for covariates; this sex-specific difference was not seen in those without CAD [69]. Another study in a multiethnic familial hypercholesterolemia (FH) cohort reported higher Lp(a) levels in women than in men with CVD, but not in those without CVD [70]. Also in FH, higher Lp(a) levels were reported among CVD-susceptible *versus* CVD-resistant women with FH [71]. The topic on Lp(a) and FH is discussed in detail by Chemello et al. in this Lp(a) review series [72]. In a longitudinal report, Lp(a) levels were significantly higher in women than in men at baseline, however, the association between elevated Lp(a) levels and 10-year first fatal/non-fatal CVD was significant in men but not in women [73]. In a large population study of Europeans, including Finns, female sex was associated with increased Lp (a) levels [74]. The studied genetic variants, as well as age, sex, and renal function, explained nearly 72% of the observed population differences in Lp(a) [74]. Among Europeans, Lp(a) levels were higher in women than in men regardless of type 2 diabetes mellitus status [75]. A further adjustment for Lp(a) levels had no impact on the HR for CVD mortality comparing men *versus* women without type 2 diabetes mellitus; however, among those with type 2 diabetes mellitus, the adjustment resulted in an increased risk in men and a decreased risk in women for CVD mortality [75]. In a recent large study of middle-aged >460,000 UK Biobank participants, Lp(a) levels were somewhat elevated in women than in men and in individuals who had established CVD at the time of enrollment [76]. While Lp(a) level predicted incident CVD in both men and women without any interaction, it was a stronger risk factor for CVD among those without diabetes mellitus than with diabetes mellitus [76]. More details on the relationship between Lp(a), diabetes mellitus, and CVD risk are provided by Lamina et al. of this Lp(a) review series [77].

Taken together, while some evidence indicates higher Lp(a) levels in females than in males, more studies are needed to establish any sex-specific differences in Lp(a) levels and relevance to CVD risk. Potential confounding effects by factors such as race/ethnicity, apo(a) size distribution, menopausal and disease status and Lp(a) measurement method should be carefully considered. Particularly, an impact of menopause on Lp(a) levels as contributory to the age-dependent relative difference between middle-aged to older men and women should be considered.

### 3.2. Hormones

#### 3.2.1. Sex hormones

Among healthy men, Lp(a) levels were not associated with endogenous testosterone, free testosterone, or sex-hormone binding globulin (SHBG) [78–80]. However, contradictory results have been reported in two studies for the association between Lp(a) levels and dehydroepiandrosterone sulfate ester (DHEA-S) [78,80], one of the most abundant endogenous androgen steroids. Among men with CAD, Lp(a) levels were significantly negatively associated with free testosterone, but not with DHEA-S [81]. In healthy postmenopausal women, inconsistent findings have been reported for the association for Lp(a) with endogenous DHEA-S or testosterone [82,83].

Exogenously administered androgens and estrogens impact Lp(a) levels. Administration of testosterone significantly reduced Lp(a) levels in healthy men [79,84–86], but not in healthy postmenopausal women [87], hypogonadal men [88] or oophorectomized women [89]. Significant reductions in Lp(a) levels were observed in perimenopausal women treated with DHEA (18%) [90], in postmenopausal osteoporotic women [91] or premenopausal women with endometriosis [92], both cases treated with stanozolol (a synthetic anabolic steroid), or in men undergoing hemodialysis treated with another anabolic steroid, nandrolone decanoate (>50% reduction at 6 months) [93]. Among male body builders, the administration of anabolic androgen steroids was associated with a lower prevalence of elevated Lp(a) levels [94] and a significant reduction in Lp(a) levels [95,96].

A large number of studies in postmenopausal women have evaluated the effects of estrogen treatment on lipids. Lp(a) levels were significantly reduced following treatments with norethisterone [97], estrogen-progestogen therapy [98], tamoxifen [99] or hormone replacement therapy (HRT) [100,101]. Lp(a) levels were significantly lower in women receiving HRT *versus* not receiving HRT in the Women Twins Study [38] and in the Women’s Health Study [102]. A meta-analysis of studies conducted during 1966–2004 quantifying the effect of HRT in postmenopausal women documented an average of 25% reduction in Lp(a) levels [103]. In Japanese women, Lp(a) levels were significantly higher in postmenopausal than in pre- or perimenopausal women and HRT reduced Lp(a) by ~19% which was retained for four years [104]. Treatment with tibolone, a synthetic steroid with weak estrogenic, progestogenic, and androgenic activity, for a year in postmenopausal women resulted in a 28% reduction in Lp(a) levels [105].

A meta-analysis based on 24 randomized controlled trials demonstrated that both HRT (mean relative difference: −20.4%) and tibolone (−25.3%) reduced Lp(a) concentrations in postmenopausal women [106]. Although the effect was statistically significant only for HRT compared to placebo or no treatment groups, there was no significant difference between HRT and tibolone regarding Lp(a) levels. Oral estrogen resulted in a greater reduction in Lp(a) concentrations than transdermal estrogen, whereas there was no significant difference comparing continuous *versus* cyclic HRT, conventional with low-dose estrogen, or estrogen monotherapy with estrogen combined with progestogen [106]. This meta-analysis concluded that HRT significantly reduces Lp(a) concentrations with oral being more effective than transdermal estradiol and that the type of HRT, dose of estrogen and addition of progestogen do not modify the Lp(a)-lowering effect of HRT [106].

#### 3.2.2. Thyroid hormones

Lp(a) levels are decreased in hyperthyroidism and increased in hypothyroidism [107]. The use of eprotirome, a liver-selective TH (thyroid hormone) analog, resulted in a dose-dependent reduction in Lp(a) concentrations (−45–55%) in statin-treated patients [108]. A similar dose-response relationship between Lp(a) reduction and eprotirome was observed in other randomized double-blind placebo-controlled trials in patients with FH [109] or with primary hypercholesterolemia [110]. Bonde et al. reported that both eprotirome and hyperthyroidism reduced concentrations of Lp(a), PCSK9, plasma cholesterol in all lipoprotein fractions, apoB and apoA-I, while cholesterol synthesis was stable [111]. TH-induced reductions in PCSK9 levels likely contributed to the lower LDL-C and Lp(a) levels in hyperthyroidism. However, significant side effects such as increases in liver enzymes and cartilage side effects in animals have been seen with eprotirome, limiting its clinical use [112]. More details on the relationship between Lp(a) and PCSK9 and its inhibition are provided by Chemello et al. of this Lp(a) review series [72].

In hypothyroidism, Lp(a) levels decreased with a 6-month levothyroxine treatment (mean ± SD: 28 ± 19 mg/dL *versus* 18 ± 11 mg/dL) in women with primary hypothyroidism (n = 12) [113]; however, levels remained elevated compared to controls (14 ± 4 mg/dL) (n = 11). In a retrospective analysis, a small increase in Lp(a) concentrations was seen after injections of recombinant human thyrotropin on a background of a stable levothyroxine dose in thyroid cancer patients who had undergone total thyroidectomy [114]. Case-control studies have found higher Lp(a) levels in patients with Hashimoto thyroiditis [115] or hypothyroidism [116] compared to healthy controls.

A recent systematic review and meta-analysis of 166 studies (23 randomized and 143 nonrandomized) conducted during 1970–2018 evaluated the impact of therapy for overt and subclinical hyper- and hypo-thyroidism on blood lipids [107]. Treatment of overt hyperthyroidism resulted in significant increases in Lp(a) by 4.18 mg/dL (95% CI: 1.65, 6.71)., TC, LDL-C, HDL-C, apoA and apoB concentrations without affecting triglycerides [107]. In contrast, no effect on lipid parameters was seen during treatment for subclinical hyperthyroidism. Levothyroxine in overt hypothyroidism significantly decreased Lp(a) by −5.6 mg/dL (95% CI:−9.06,−2.14) and induced moderate to large reductions in TC, LDL-C, HDL-C, triglycerides, apoA1, and apoB concentrations. Levothyroxine in subclinical hypothyroidism showed similar changes but with a smaller magnitude. A recent study reported elevated Lp(a) levels in patients with overt (n = 280) or subclinical (n = 272) hypothyroidism compared to healthy controls (n = 270) [117].

#### 3.2.3. Growth hormones

Growth hormone (GH) replacement therapy increases Lp(a) levels. Among adults with adult-onset pituitary insufficiency, Lp(a) levels increased markedly during GH treatment and were about twice as high compared with pre-treatment levels [118]. Among adults with postoperative GH deficiency, recombinant human GH treatment increased significantly Lp(a) levels at 12 months posttreatment, independently of baseline Lp(a) levels and apo(a) isoforms [119]. More recently, a prospective observational study demonstrated that a GH replacement therapy in men with GH deficiency resulted in a significant increase in Lp(a) levels (mean: from 27.4 nmol/L to 34.3 nmol/L) [120]. There were no correlations between baseline Lp(a) levels (or the increase) and concentrations of TH or insulin-like growth factor-1 [120].

## 4. Pathologies that modify Lp(a) concentrations

### 4.1. Kidney diseases

The role of kidney diseases in impacting Lp(a) levels has been the subject of many studies. The effects have varied depending on the specific condition and disease stage, the amount of proteinuria, or treatment modalities. In patients with severe chronic kidney disease (CKD), i. e., end-stage renal disease (ESRD), Dieplinger et al. observed higher Lp (a) levels compared with healthy controls despite similar apo(a) isoforms distribution in both groups [121]. In a diverse group of CKD patients, Milionis et al. found significantly elevated Lp(a) levels in patients with mild to moderate chronic renal failure (CRF) and patients treated with hemodialysis (HD) or continuous ambulatory peritoneal dialysis (CAPD) compared with controls, a finding not explained by the apo(a) size variability [122]. In another study among CRF patients, Lp(a) levels were twice as high as in healthy controls and were influenced by nutritional status [123]. Addressing a void regarding the changes in Lp (a) levels in early stages of kidney impairment, Kronenberg et al. conducted a detailed assessment of the relationship between Lp(a) levels, apo(a) sizes, and kidney function in 227 patients with non-nephrotic kidney disease (NNKD) with various stages of kidney impairment [124]. The results confirmed higher Lp(a) levels in patients with NNKD compared with healthy controls. Of note, the median Lp(a) levels increased as the kidney function impaired (11.0 at GFR >90, 18.4 at GFR 45–90 and 24.4 mg/dL at GFR <45 mL/min/1.73 m^2^). These findings suggested that Lp(a) levels begin to increase even in early stages of kidney impairment [124,125] and showed an inverse association between Lp(a) levels and kidney function [125,126].

**Fig. 3.**
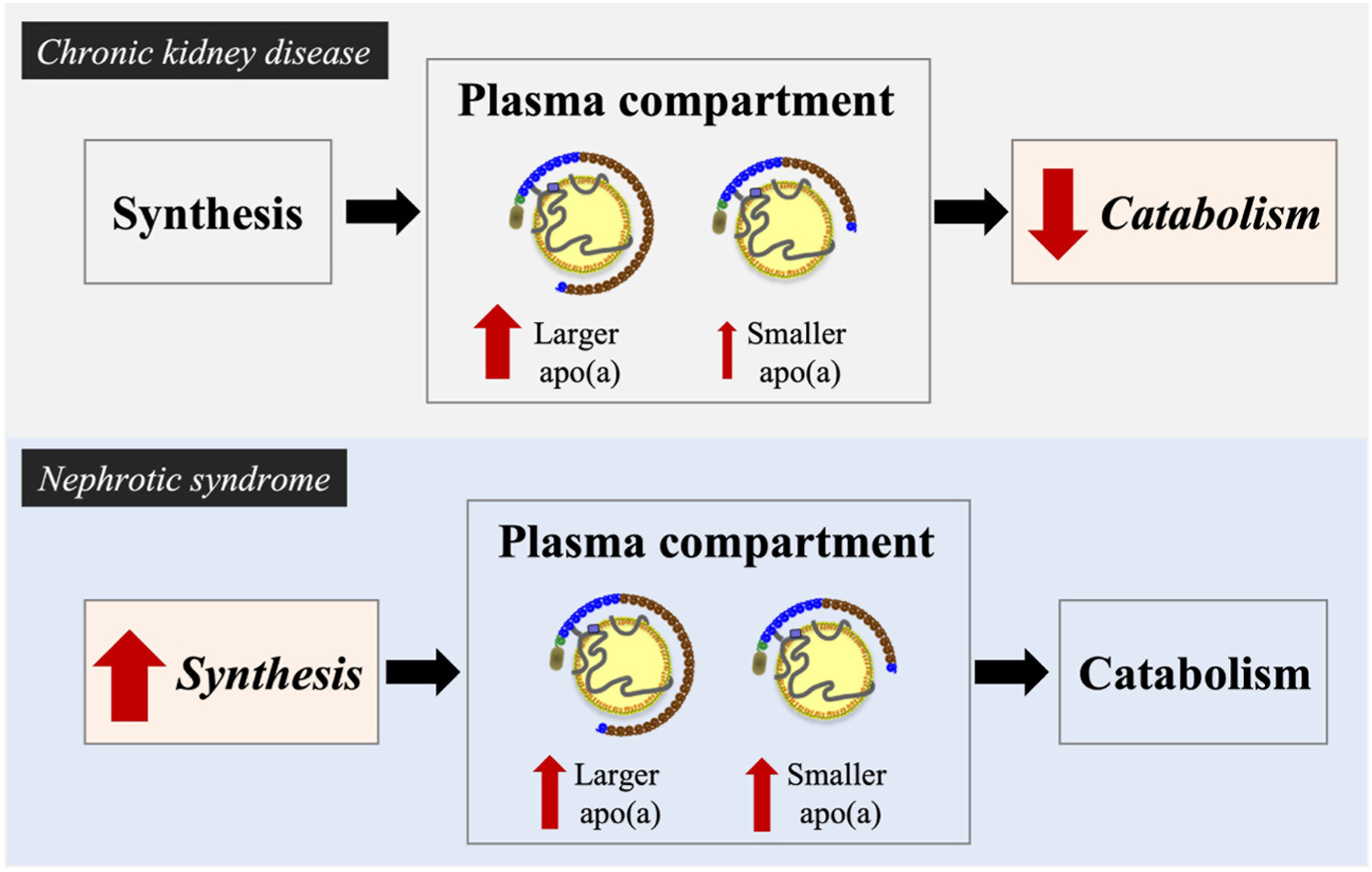
Differences underlying increased Lp(a) levels in chronic kidney disease *versus* nephrotic syndrome in relation to homeostasis and genetically determined apolipoprotein(a) sizes. Kidney diseases influence Lp(a) levels. In patients with chronic kidney disease (upper panel), Lp(a) catabolism is decreased, resulting in apo(a)-phenotype specific increases in Lp(a) levels. Thus, the increase is largely due to increases in the large apo(a) isoform associated levels. In contrast, in patients with nephrotic syndrome (lower panel), Lp(a) synthesis is increased, resulting in simultaneous increases for both large and small apo(a) size associated levels.

A common finding among CKD patients has been that the increase in Lp(a) levels varies across apo(a) sizes as only patients with large size apo (a) isoforms exhibited a 2- to 4-fold higher Lp(a) level compared with controls [121] (Fig. 3). When compared with apo(a) phenotype-matched controls, the significant association between Lp(a) levels and kidney function was seen in patients with large apo(a) isoforms, but not in patients with small isoforms [124]. Thus, median Lp(a) levels in patients with large apo(a) isoforms were 6.2 mg/dL at GFR >90, 14.2 mg/dL at GFR 45–90, and 18.0 mg/dL at GFR <45 mL/min/1.73 m^2^, all of which were markedly elevated compared with the median level of 4.4 mg/dL in controls. Other studies have shown that the Lp(a) response was dependent on apo(a) sizes also during dialysis treatment. Apo(a) size specific increases in Lp(a) levels were seen among patients with NNKD or ESRD patients treated with HD [121,126,127]. Thus, Lp(a) levels were higher in HD patients compared with healthy controls (13.6 *versus* 9.2 mg/dL) as was the prevalence of a high Lp(a) level (23% *versus* 12%), despite a similar distribution of apo(a) isoforms in both groups [127]. Again, this rise in Lp(a) level in HD patients *versus* controls was limited to large apo (a) isoform group only (14 *versus* 8 mg/dL) and was associated with heightened inflammation [127]. More details on the association of Lp(a) with inflammation are provided by Dzobo et al. of this Lp(a) review series [128].

While the findings for HD have been consistent, some variability is reported for CAPD. In a large multicenter study of ESRD patients, Kronenberg et al. reported elevated Lp(a) levels in CAPD patients compared to HD patients (34.6 *versus* 23.4 mg/dL), while both were significantly higher compared to those of healthy controls (18.4 mg/dL) [126]. The higher Lp(a) levels in both patient groups (*versus* controls) were not explained by apo(a) size variability as all three groups had a similar frequency of small apo(a) isoforms. Lp(a) levels were significantly elevated for the large apo(a) isoforms in both patient groups (HD and CAPD) compared with controls [126]. Of note, CAPD patients had significantly higher Lp(a) levels than did the HD patients for large apo(a) isoforms (26.1 *versus* 17.2 mg/dL) [126]. However, an increase in Lp(a) levels for patients with small apo(a) isoforms also been reported among CAPD patients. Thus, in contrast to HD, CAPD has been associated with elevated Lp(a) levels regardless of apo(a) sizes [122]. Furthermore, a study in children treated with peritoneal dialysis reported higher Lp(a) levels compared to matched controls, but no apo(a) size data was available [129]. On the other hand, some studies have found no relationship between Lp(a) levels, GFR and/or apo(a) isoforms. For example, in the Modification of Diet in Renal Disease Study enrolled 804 patients with CKD (stages 3–4 with a GFR range of 13–55 mL/min/1.73 m^2^), Lp(a) level was not associated with GFR [130]. Among kidney donors whose GFR was reduced by ~36% at 1 year post donation *versus* before donation, Lp(a) was not changed [131].

The higher Lp(a) level in CKD patients seen in many reports has stimulated studies of underlying mechanisms. *In vivo* turnover studies using stable isotopes in HD patients suggested that a reduced catabolic rate of Lp(a)-apoB and apo(a) was responsible for the Lp(a) elevation in CKD [132]. Given the differential increase in Lp(a) depending on apo(a) sizes, this finding brings up the interesting possibility that CKD might affect Lp(a) catabolism differently depending on apo(a) size properties. Thus, metabolic studies under CKD conditions taking apo(a) size into account could offer valuable insights into Lp(a) metabolic properties.

In contrast to CKD conditions, pronounced increases in Lp(a) levels occur in all apo(a) size groups in patients with nephrotic syndrome (NS) [125,133–135]. Wanner et al. demonstrated that Lp(a) levels were increased in patients with NS (diabetic and non-diabetic) compared with controls across the apo(a) size range [134]. Moreover, a large decrease in Lp(a) levels was seen in non-diabetic NS patients following remission of the syndrome with immunosuppressive therapy [134]. Similarly, Kronenberg et al. reported ~5-fold elevated Lp(a) levels in patients with non-diabetic NS compared with controls [135]. While the increase was partly explained by a different distribution of apo(a) size phenotypes in the patient group *versus* the control group, both small (40–75%) and large (100–500%) apo(a) isoforms were associated with significantly elevated Lp(a) levels in the patient group. Others have also found significantly higher Lp(a) levels in patients with NS (severe proteinuria) or chronic glomerulonephritis (moderate proteinuria) compared with healthy controls [136] and a decrease in Lp(a) levels with the remission of the syndrome [137]. Shedding light into mechanisms underlying the increased Lp(a) level in NS patients, a turnover study by van der Velden et al. [138] showed that while the fractional catabolic rate of Lp(a) was comparable between NS patients and controls, the absolute synthesis rate of Lp(a) correlated with Lp(a) concentration in all participants. These data suggest a role for an increased synthesis, rather than a decreased catabolism, as a cause for elevated Lp(a) levels in NS. It has been proposed that in NS and probably also in CAPD, patients lose a significant amount of proteins by urine and dialysate, respectively, that the increased synthesis of Lp(a) might be a result of a counteraction to keep up the oncotic pressure and/or viscosity of blood [136,138,139] (Fig. 3).

Renal transplantation results in significant reductions in Lp(a) levels consistent with the acquired nature of the Lp(a) abnormality [125,139]. Prospective studies with variable follow-up periods have shown substantial decreases in Lp(a) levels [140–143]. The decrease was observed only in patients with large apo(a) isoforms [140] or linked to a reduced expression of large apo(a) isoforms [141]. Rosas et al., observed a rapid decline in Lp(a) levels after renal transplantation reaching a 35% reduction at 2 weeks [143]. Each reduction of 50% in creatinine was associated with ~11% reduction in Lp(a) levels. Among patients with a relapse and worsening kidney function, a marked increase of the large isoform-associated Lp(a) levels was noted [141]. Consistent with the reports of higher Lp(a) levels in CAPD compared to HD, Kerschdorfer et al. found a large decrease post transplantation in CAPD-*versus* HD-treated patients [142]. Similarly, a larger decrease was seen among patients with higher Lp(a) levels before renal transplantation or patients with large apo(a) isoforms [142]. In contrast, variable results regarding Lp(a) have been observed in cross-sectional studies [144–149].

The influence of immunosuppressive therapies on Lp(a) levels in renal transplant recipients has also been explored. Higher Lp(a) levels have been reported in recipients treated with cyclosporin *versus* azathioprine or prednisolone [150–152] independently of apo(a) size variability [150], while others have found no evidence for a role of immunosuppressive therapy [140,142,146,149,153,154]. A retrospective analysis showed that young (<35 years old) renal transplant recipients with small apo(a) isoforms had a significantly shorter long-term graft survival compared with those with large apo(a) isoforms, independent of the number of HLA mismatches, sex, or immunosuppressive therapy [155]. Overall, whether the reduction in Lp(a) levels after renal transplantation is influenced by immunosuppressive therapies remains to be seen [139,156].

In summary, there is strong evidence to support a role of the kidney in impacting Lp(a) levels. Both the increase in Lp(a) levels in CKD and the decrease in Lp(a) levels after renal transplantation are likely related to the degree of kidney function impairment. In contrast, the increase in Lp(a) levels in NS appears to result from an increased production in response to proteinuria [139]. The potential roles of additional factors in influencing Lp(a) in CKD remain to be determined.

### 4.2. Liver diseases

As the concentration of Lp(a) is primarily regulated by the hepatic apo(a) synthetic rate, liver diseases have the potential to influence Lp(a) levels. In general, hepatocellular damage is associated with reduced Lp (a) levels, where the decrease in levels is in parallel with the disease progression [157–159]. Patients with liver cirrhosis [160] and hepatitis [157,161,162] exhibited lower Lp(a) levels compared to healthy controls. Geiss et al. [162] observed a 41% reduction in Lp(a) level, independent of apo(a) isoform size, in patients with acute hepatitis A, B and C (HCV). Lp(a) levels were significantly lower in HCV core protein-positive patients compared to core-negative cases [163]. A significant increase in Lp(a) levels was seen in chronic active HCV patients with a complete response to a 6-month interferon treatment [157]. Also in patients with chronic HCV (81% cirrhotic), where the majority (93%) achieved a sustained virological response with a 24-week direct acting antiviral treatment, Lp(a) levels rose by ~2-fold [164].

Studies in patients with nonalcoholic steatohepatitis (NASH) or non-alcoholic fatty liver disease (NAFLD) have shown variable results with regard to Lp(a). A study on NASH showed similar Lp(a) levels to those of healthy controls [165]. Several recent Asian population studies have reported on the association between Lp(a) levels and different stages of NAFLD. Among Korean adults, Lp(a) levels decreased with the severity of NAFLD and the prevalence of NAFLD decreased with the Lp(a) tertiles [166]. The inverse association between Lp(a) levels and NAFLD remained significant after multivariate adjustment, but was attenuated when taking insulin resistance into account [166]. A large cross-sectional study in Korean adults confirmed an inverse association of Lp(a) levels with NAFLD with significantly lower levels in the NAFLD group *versus* the control group [167]. The odds ratio for NAFLD was the lowest in the top Lp(a) quartile [167]. Among Japanese patients with biopsy-confirmed NAFLD, Lp(a) levels were lower in patients with advanced fibrosis and an inverse association between the advanced fibrosis, NASH and Lp(a) levels remained significant in multivariate models [168]. In contrast, in Chinese patients with NAFLD, concentrations of Lp(a) and liver enzymes increased with the disease severity [169]. The odds ratio of Lp(a) levels for NASH was 1.61 and a combination of Lp(a) and liver enzymes improved the prediction for NASH [169]. Among Malaysians, a recent cross-sectional study in a high CVD risk cohort (patients with obstructive sleep apnea) found 3.5-fold higher Lp(a) levels in patients with NAFLD compared with those without NAFLD [170]. A stepwise increase in Lp(a) levels as well as in carotid intima media thickness was observed with a worsening clinical condition [170]. The differences underlying these heterogenous associations between Lp(a) levels and NAFLD across population groups need to be elucidated in future studies using standardized measurement methodology as well as potential impact from accompanying metabolic conditions, age, gender and genetics.

## 5. Conclusions

The current evidence on non-genetic influences on Lp(a) concentration indicates a potential role for diet, hormones and liver and kidney diseases (Box 1). In particular, strong consistent evidence suggests an impact on Lp(a) concentration by reducing dietary saturated fat intake, sex hormones and hormone replacement therapies and kidney diseases and treatment modalities (Table 1). In contrast, more data is needed to firmly establish any potential role for PA/exercise and certain liver diseases in influencing Lp(a) concentration. The use of wellstandardized assay methods for Lp(a) measurement is of paramount importance for studying non-genetic influences on Lp(a) as discussed by a further review of this series [171]. Additional factors of consideration include large sufficiently powered sample sizes and potential confounders, including but not limited to, race/ethnicity, metabolic status and genetic variability. Research to elucidate mechanisms underlying the changes in Lp(a) concentration and the modulation of Lp(a) properties beyond its plasma level will help improve our understanding of non-genetic influences on Lp(a). Finally, the clinical significance of the changes in Lp(a) concentration and its risk mediating properties due to non-genetic factors, including lifestyle interventions, remains to be seen.

## Figures and Tables

**Table 1:** A broad summary of non-genetic factors that may influence Lp(a) concentrations described in this review article.

	Interventions and conditions	Association with Lp(a) concentration [Reference]
**1**	**Diet**	
	a. Replacement of dietary saturated fats with carbohydrate or unsaturated fats	~8–20% increase [7–12,36]
	b. Low-carbohydrate, high-saturated fat diet	~15% decrease [18,36]
	c. Diets enriched with walnuts or pecans	~6–15% decrease [26,27,36]
	d. Alcohol consumption	No association or minor decrease [32–35]
**2**	**Physical activity and & exercise**	No or minimal association [46–50]
**3**	**Sex, hormones** and **associated conditions**	
	a. Sex	No association or higher levels in females than males [61–63,67,73–76]
	b. Sex hormones (endogenous)	No or minor association [78–83]
	c. Postmenopausal hormone replacement therapy (HRT)	~20–25% decrease; a greater decrease with oral *vs* transdermal estrogen; no difference between continuous *vs* cyclic HRT [103,106]
	d. Hyperthyroidism	Decreased Lp(a); treatment of overt hyperthyroidism increased Lp(a) by 20–25% [107,111]
	e. Hypothyroidism	Elevated Lp(a); treatment of overt and subclinical hypothyroidism decreased Lp(a) by 5–20% [107,113,116,117]
	f. Growth hormone replacement therapy	~25–100% increase [118–120]
**4**	**Chronic kidney disease**	
	a. Chronic kidney disease and hemodialysis	Elevated Lp(a); an inverse association with kidney function; a 2–4-fold higher level only in patients with large size apo(a) *vs* controls [121,122,124–127]
	b. Continuous ambulatory peritoneal dialysis	~2-fold elevated *vs* controls [122,126]
	c. Nephrotic syndrome	~3–5-fold increase compared to controls [125,133–135]
	d. Kidney transplantation	Significant reduction; near normalization [125,139–143]
**5**	**Liver disease**	
	a. Hepatocellular damage	Decreased in parallel with the disease progression; >40% reduction in hepatitis; a 2-fold increase with antiviral treatment [157–164]
	b. Non-alcoholic fatty liver disease	Inconsistent association across population groups [166–170]

## Key points box 1 Non-genetic influences on Lp(a) concentrations.

- While Lp(a) is under strong genetic regulation, a number of other factors, including some clinical conditions, influence levels.
- Replacement of dietary saturated fat with protein, carbohydrates or unsaturated fat increases Lp(a) levels in the order of 10–15%.
- Modulation of physical activity has not been shown to consistently affect Lp(a) levels.
- In contrast to endogenous sex hormone levels, under non-pregnant conditions, exogenously administered androgens and estrogens impact Lp (a) levels.
- Both hyper- and hypothyroid conditions modestly impact Lp(a) levels.
- Lp(a) levels increase in chronic kidney disease and nephrotic syndrome – in the former, the increase is primarily limited to Lp(a) with larger size apo(a) isoforms. Baseline Lp(a) levels are largely restored after renal transplantation.
- Lp(a) levels are associated with hepatocellular damage – a decrease is seen in relation to disease progression. Whether Lp(a) is influenced by non-alcoholic fatty liver disease remains to be clarified.
